# 

**Published:** 1898-03

**Authors:** 


### BOOKS RECEIVED.

A Text-book on Surgery, general, operative and mechanical. By John A. Wyeth, M. D., Professor of Surgery in, and President of the Faculty of the New York Polyclinic Medical School and Hospital; late Surgeon to Mt. Sinai Hospital and Consulting Surgeon to St. Elizabeth's Hospital, etc., etc. Third edition, revised and enlarged. Royal 8vo, pp. x—997. New York: D. Appleton & Co. 1898.

The American Year-Book of Medicine and Surgery; being a yearly digest of scientific progress and authoritative opinion in all branches of medicine and surgery, drawn from journals, monographs and text-books of the leading American and foreign authors and investigators. Collected and arranged with critical editorial comments by eminent American specialists and teachers. Under the general editorial charge of George M. Gould, M. D. One volume of nearly 1,200 pages, profusely illustrated with numerous wood-cuts in text, and thirty-three half-tone and colored plates. Prices: cloth, $6.50 net; half-morocco, $7.50 net. Philadelphia: W. B. Saunders. 1898.

A System of Practical Medicine. By American authors. Edited by Alfred Lee Loomis, M. D., late Professor of Pathology and Practical Medicine in the New York University, and William Gilman Thompson, M. D., Professor of Medicine in the New York University. To be completed in four imperial 8vo volumes, containing from 900 to 1,000

pages each, fully illustrated in colors and in black. Volume III.—Diseases of the Alimentary Canal, Peritoneum, Liver and Gall Bladder, Spleen, Pancreas and Thyroid Gland, Chronic Metal Poisoning, Alcoholism, Morphinism, Infectious Diseases Common to Man and Animals, Miscellaneous subjects. For sale by subscription. Per volume, cloth, $5.00; leather, $6.00; half morocco, $7.00. Philadelphia and New York: Lea Brothers & Co., Publishers. 1898.

Sexual Neurasthenia: Its Hygiene, Causes, Symptoms and Treatment, with a chapter on Diet for the Nervous. By George M. Beard, A. M., M. D., formerly Lecturer on Nervous Diseases in the University of the City of New York; Fellow of the New York Academy of Medicine, etc., etc. Edited, with notes and additions, by A. D. Rockwell, A. M., M. D., formerly Professor of Electro-Therapeutics in the New York Post-Graduate Medical School and Hospital, etc., etc. With formulas. Fifth edition. Octavo, pp. xii.—308. New York. E. B. Treat & Co. 1898.

Transactions of the Texas State Medical Association. Twenty-ninth Annual Session, held at Paris, Texas, April 27, 28, 29 and 30, 1897. H. A. West, M. D., Secretary, Galveston, Texas.

Transactions of the Medical Society of the State of North Carolina. Forty-fourth Annual Meeting held at Morehead City, N. C., June 8, 9 and 10, 1897. R. D. Jewett, M. D., Secretary, Wilmington, N. C.

Lexique-Formulaire des Nouveautés Médicales, par le professeur Paul Lefert, 1 vol. in–18 de 336 pages, cartonné, 3 fr. Librairie J. B. Baillière et fils, 19, rue Hautefeuille (près du boulevard Saint-Germain), à Paris.

Orthopedic Surgery. By James E. Moore, M. D., Professor of Orthopedia and of Clinical Surgery in the College of Medicine of the University of Minnesota; Fellow of the American Surgical Association; Member of the American Orthopedic Association; Surgeon to St. Barnabas Hospital, Minneapolis, etc. Octavo volume of 360 pages, illustrated with 177 half-tones, from photographs made specially for this work. Price, cloth, $2.50 net. Philadelphia: W. B. Saunders, 925 Walnut street. 1898.

The Diseases and Injuries of the Conjunctiva, especially the so-called Granulated Lids. By John H. Thompson, M. D., Professor of Ophthalmology and Otology, Kansas City Medical College, Kansas City, Mo. Illustrated. Kansas City, Mo.: Hudson-Kimberly Publishing Co. 1897.

